# Elevated BCAA catabolism reverses the effect of branched-chain ketoacids on glucose transport in mTORC1-dependent manner in L6 myotubes

**DOI:** 10.1017/jns.2024.66

**Published:** 2024-10-18

**Authors:** Gagandeep Mann, Olasunkanmi A. John Adegoke

**Affiliations:** School of Kinesiology and Health Science and Muscle Health Research Centre, York University, Toronto, ON, Canada

**Keywords:** Branched-chain amino acids, Insulin resistance, Metabolism, mTORC1, Skeletal muscle

## Abstract

Plasma levels of branched-chain amino acids (BCAA) and their metabolites, branched-chain ketoacids (BCKA), are increased in insulin resistance. We previously showed that ketoisocaproic acid (KIC) suppressed insulin-stimulated glucose transport in L6 myotubes, especially in myotubes depleted of branched-chain ketoacid dehydrogenase (BCKD), the enzyme that decarboxylates BCKA. This suggests that upregulating BCKD activity might improve insulin sensitivity. We hypothesised that increasing BCAA catabolism would upregulate insulin-stimulated glucose transport and attenuate insulin resistance induced by BCKA. L6 myotubes were either depleted of BCKD kinase (BDK), the enzyme that inhibits BCKD activity, or treated with BT2, a BDK inhibitor. Myotubes were then treated with KIC (200 μM), leucine (150 μM), BCKA (200 μM), or BCAA (400 μM) and then treated with or without insulin (100 nM). BDK depletion/inhibition rescued the suppression of insulin-stimulated glucose transport by KIC/BCKA. This was consistent with the attenuation of IRS-1 (Ser612) and S6K1 (Thr389) phosphorylation but there was no effect on Akt (Ser473) phosphorylation. The effect of leucine or BCAA on these measures was not as pronounced and BT2 did not influence the effect. Induction of the mTORC1/IRS-1 (Ser612) axis abolished the attenuating effect of BT2 treatment on glucose transport in cells treated with KIC. Surprisingly, rapamycin co-treatment with BT2 and KIC further reduced glucose transport. Our data suggests that the suppression of insulin-stimulated glucose transport by KIC/BCKA in muscle is mediated by mTORC1/S6K1 signalling. This was attenuated by upregulating BCAA catabolic flux. Thus, interventions targeting BCAA metabolism may provide benefits against insulin resistance and its sequelae.

## Introduction

Branched-chain amino acids (BCAA; leucine, valine, isoleucine) stimulate muscle protein synthesis, and regulate body weight and glucose homeostasis.^(1)^ On the other hand, increased circulating levels of BCAA and BCAA metabolites, branched-chain α-ketoacids (BCKA: α-ketoisocaproic acid (KIC), α-keto-β-methylvaleric acid (KMV), α-ketoisovaleric acid (KIV)) are seen in insulin-resistant states like type 2 diabetes mellitus (T2DM).^(2–5)^ Also, BCAA and their metabolites induce insulin resistance.^(5–10)^ BCAA increase the activation of mammalian/mechanistic target of rapamycin complex 1 (mTORC1) and p70 ribosomal protein S6 kinase-1 (S6K1). mTORC1/S6K1 activation results in the inhibitory phosphorylation of serine residues of insulin receptor substrate-1 (IRS-1) (Ser636, Ser312, Ser616 in humans, and Ser632, Ser307 and Ser612 in mice)^(11,12)^ by S6K1. Consequently, this leads to the degradation of IRS-1^(13)^ and thus inhibiting downstream insulin signalling.^(14,15)^ Additionally, insulin-resistant states can regulate the BCAA catabolic pathway and the enzymes involved,^(16–19)^ and targeting this pathway can potentially improve insulin sensitivity.^(16,19)^

BCAA are transaminated by branched-chain aminotransferase 2 (BCAT2) predominantly in skeletal muscle, producing BCKA. The BCKA are then oxidatively decarboxylated, especially in the liver where the activity of the enzyme responsible, branched-chain ketoacid dehydrogenase (BCKD), is high.^(20)^ Thus, most studies investigate BCKD and its activity in the liver. However, depletion of the E1 alpha subunit of BCKD in L6 myotubes reduced insulin-stimulated glucose transport,^(6)^ suggesting a connection between skeletal muscle BCAA catabolism and insulin signalling. On the other hand, increasing or decreasing skeletal muscle BCAA oxidation had no effect on whole-body insulin sensitivity in mice.^(21)^ This is interesting as increasing BCKD activity with pharmacological agents in rodents^(19,21–23)^ and humans^(24)^ provides benefits against insulin resistance. Since skeletal muscle insulin resistance is the primary defect in type 2 diabetes,^(25)^ it is imperative to analyse how BCKD activity in skeletal muscle affects insulin sensitivity. Here, using myotubes treated with different mixes of BCAA/BCKA, we investigated the effect of upregulating BCAA catabolism at the level of BCKD on measures of insulin action. In this context, we also examined whether alterations to mTORC1 activity interact with BCAA catabolic pathway to regulate insulin actions in myotubes.

## Methods

### Reagents

Alpha modification of Eagle’s medium (AMEM, #310-010-CL), phosphate-buffered saline (PBS, #311-010-CL), trypsin (#325-043-CL), and antibiotic–antimycotic (ab-am, #15240-062) preparations were purchased from Wisent (St Bruno, Quebec, Canada). Foetal bovine serum (FBS, #12483-020), horse serum (HS, #26050088), Lipofectamine RNAiMAX (#13778-150), and Opti-MEM 1X Reduced Serum Medium (#31985070) were purchased from Thermo Fisher Canada (Burlington, Ontario Canada). Amino acid-free Roswell Park Memorial Institute (RPMI) 1640 medium (R8999-04A) was purchased from US Biologicals (Salem MA). L-leucine (#L8912), L-isoleucine (#I7403), L-valine (#V0513), sodium 4-methyl-2-oxovalerate (sodium salt of KIC, #K0629), 3-methyl-2-oxovaleric acid (sodium salt of KMV, #K7125), 3-methyl-2-oxobutyrate (sodium salt of KIV, #198994), protease (#P8340) and phosphatase (#P5726) inhibitor cocktails, anti-gamma tubulin antibody (#T6557), siRNA oligonucleotides, amino acid standard (#AAS-18), dimethyl sulfoxide (DMSO) (#D5879-100ML), O-phthalaldehyde (OPA, #P1378), 4,6-dimorpholino-*N*-(4-nitrophenyl)-1,3,5-triazin-2-amine (MHY1485, #SML0810), 1,2-diamino-4,5-methylenedioxybenzene (DMB, #66807), trypan blue dye (#T8154), and 3,6-dichlorobenzo[b]thiophene-2-carboxylic acid (BT2, #592682) were purchased from Sigma Aldrich (Oakville, Ontario, Canada). Rapamycin (#Rap004) was purchased from BioShop (Burlington, Ontario, Canada). Antibodies against phosphorylated (ph)-S6K1 (Thr389, #9234, 1:1000), ph-S6 (Ser235/6, #4858, 1:2000), ph-Akt (Ser473, #4060, 1:1000), ph-BCKDH-E1α (Ser293, #40368, 1:1000), ph-ACC (Ser 79, #3661, 1:1000) and ph-IRS-1 (Ser612, #3203, 1:1000), as well as horseradish peroxidase (HRP)-conjugated anti-rabbit (#7074, 1:10000) and anti-mouse (#7076, 1:10000) secondary antibodies were purchased from Cell Signaling Technology (Danvers, MA). Antibody against BCAT2 (#16417-1-AP) was purchased from ProteinTech (Rosemont, IL). Antibody against BDK (#PA5-31455) and Pierce BCA protein assay kit (#23225) were purchased from Thermo Fisher Canada (Burlington, Ontario, Canada). [^3^H]-2-deoxyglucose (#NET549) was purchased from Perkin Elmer (Markham, Ontario, Canada), U-^14^C labelled valine (ARC-0678) was purchased from American Radiolabeled Chemicals, while chemiluminescence substrate (#WBKLS0500) was from Millipore (Etobicoke, Ontario, Canada). L6 rat skeletal muscle myoblasts (#CRL-1458) were purchased from American Type Culture Collection (Manassas, VA).

### Cell culture

L6 rat skeletal muscle cells were cultured in 10-cm plates with growth medium (GM: AMEM supplemented with 10% FBS and 1% antibiotic-antimycotic preparations). Cells were seeded (2 × 10^5^cells/well) in 6-well plates for western blot experiments or (10^5^ cells/well) in 12-well plates for glucose transport experiments. They were allowed to proliferate for 48 h or until they became 90–100% confluent. They were then shifted into the differentiation medium (DM: AMEM, 2% HS, 1% antibiotic-antimycotic preparations) and replenished with fresh DM every 48 h. Myotubes were used on day 5 (D5) of differentiation.

### BCAA and BCKA treatment

**Fig. 1.**
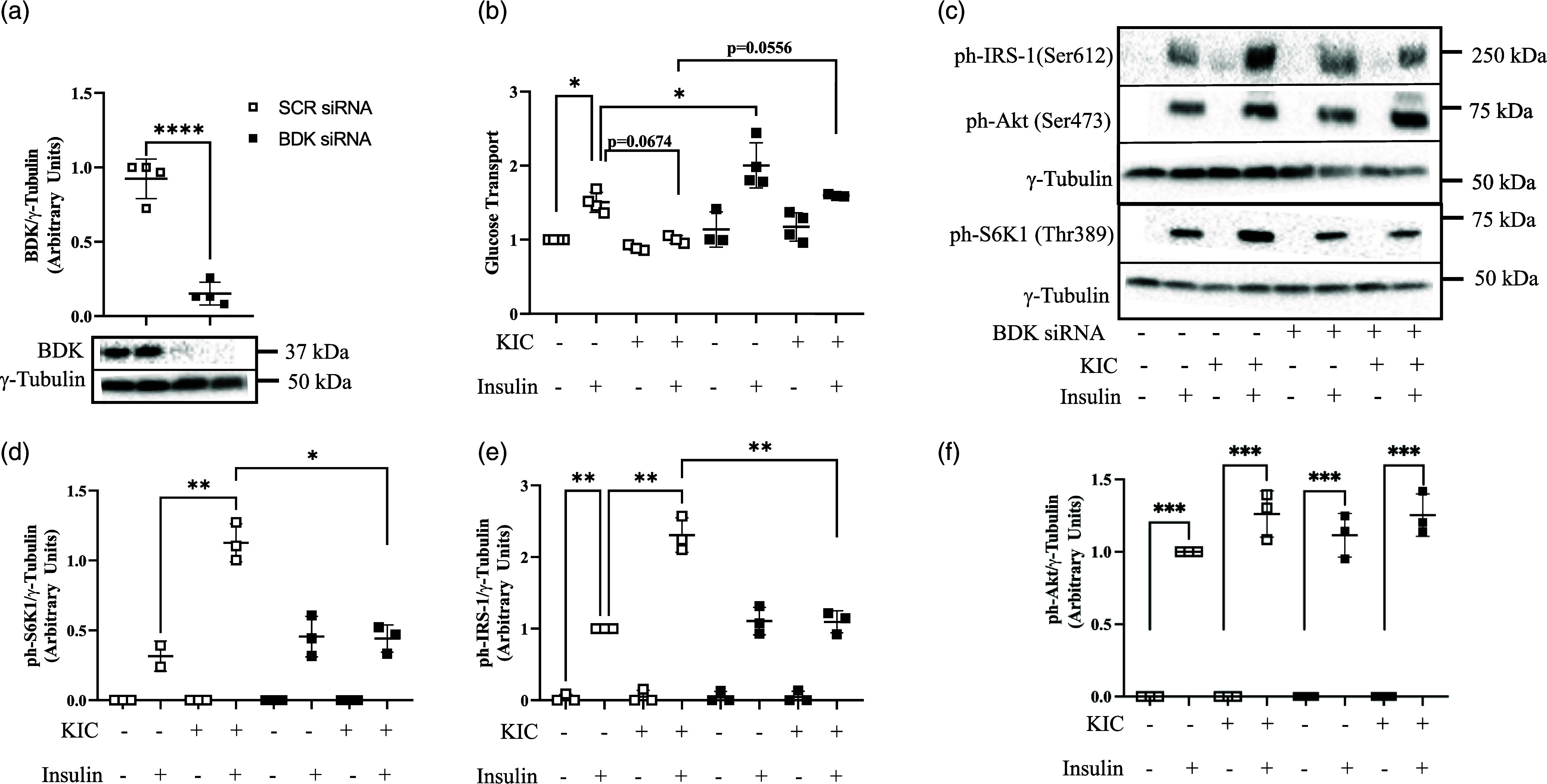
BDK depletion attenuates the suppressive effect of KIC on insulin-stimulated glucose transport and the activation of S6K1/IRS-1. L6 myotubes were transfected with control (SCR) or BDK siRNA oligonucleotides. Forty-eight h later, cells were starved in a medium lacking amino acids and serum for 3 h. They were then treated without (−KIC) or with 200 μM KIC (+KIC) for 30 min. After, cells were incubated with or without 100 nM insulin for 20 min. Proteins in lysates were immunoblotted against BDK (a). Glucose transport assay was performed (b). Proteins in lysates were also immunoblotted against ph-S6K1^Thr389^ (c, d), ph-IRS-1^Ser612^ (c, e), ph-Akt^Ser473^ (c, f). Proteins for western blot were normalised to γ-tubulin as the loading control. Glucose transport was normalised to the no insulin (–insulin) group in the SCR condition. n = 3–4 biological replicates with 3 technical replicates per experiment. Data are presented as Means ± SD * P < 0.05, ** P < 0.01, *** P < 0.001, **** P < 0.0001.

**Fig. 2.**
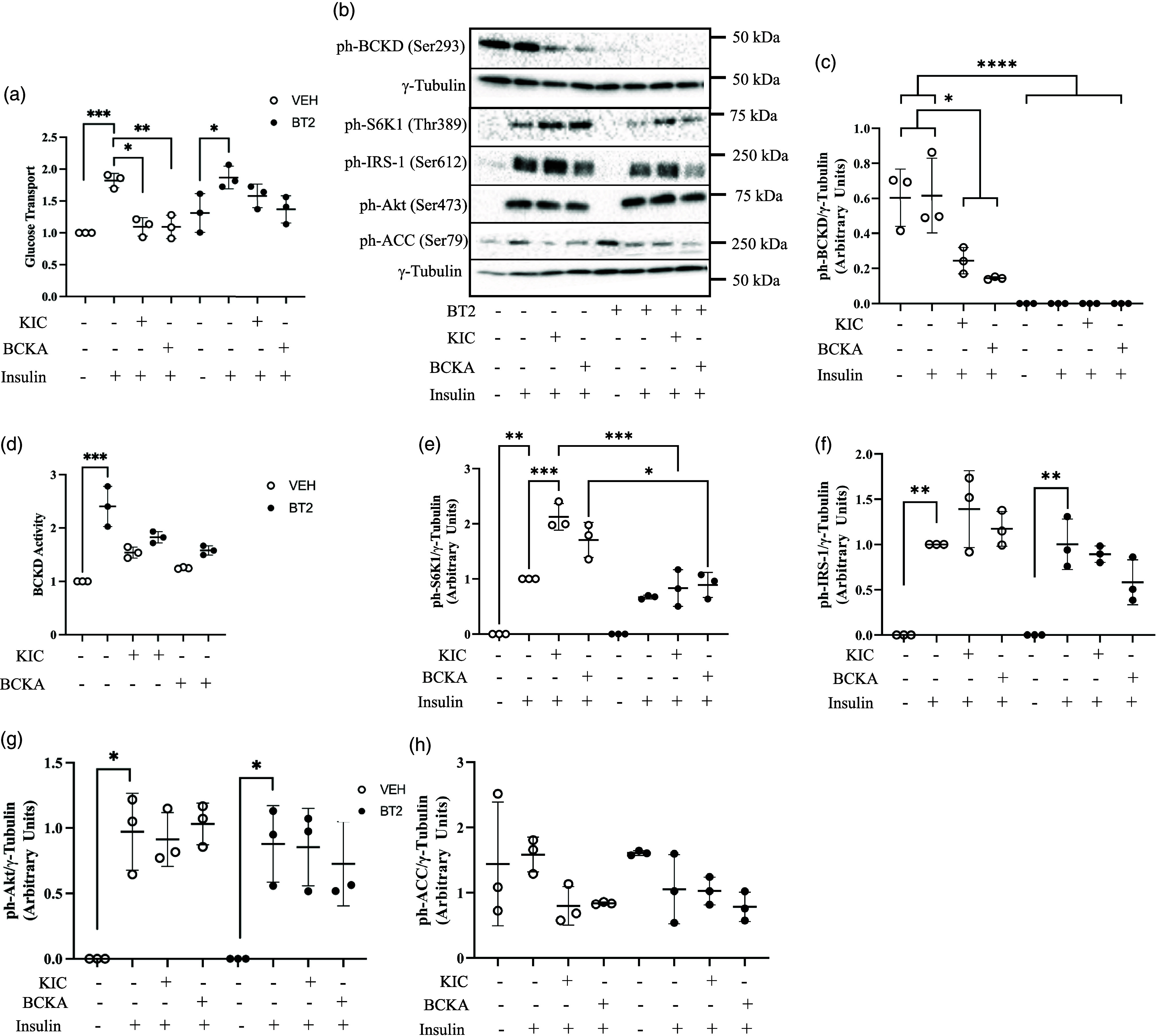
BDK inhibition attenuates the suppression of glucose transport by BCKA. L6 myotubes were incubated for 3 h in 250 μM of BT2 in a starvation medium that lacked amino acids and serum. Incubation then continued in starvation medium with BT2 along with the addition of KIC (200 μM) or BCKA (total 200 μM: consisting of 76 μM of KIC and 62 μM for each of KMV and KIV) for 30 min. After, cells were incubated with or without 100 nM insulin for 20 min. Glucose transport assay (a) was then performed. Protein in lysates were immunoblotted against ph-BCKD^Ser293^ (b, c). BCKD activity assay was performed (d). Protein in lysates were also immunoblotted against ph-S6K1^Thr389^ (b, e), ph-IRS-1^Ser612^ (b, f), ph-Akt^Ser473^ (b, g), and ph-ACC^Ser79^ (b, h). Proteins for western blot were normalised to γ-tubulin as the loading control. Glucose transport was normalised to the no insulin (–insulin) group in the vehicle (VEH) condition. n = 3 biological replicates with 3 technical replicates per experiment. Data are presented as Means ± SD. * P < 0.05, ** P < 0.01, *** P < 0.001, **** P < 0.0001.

**Fig. 3.**
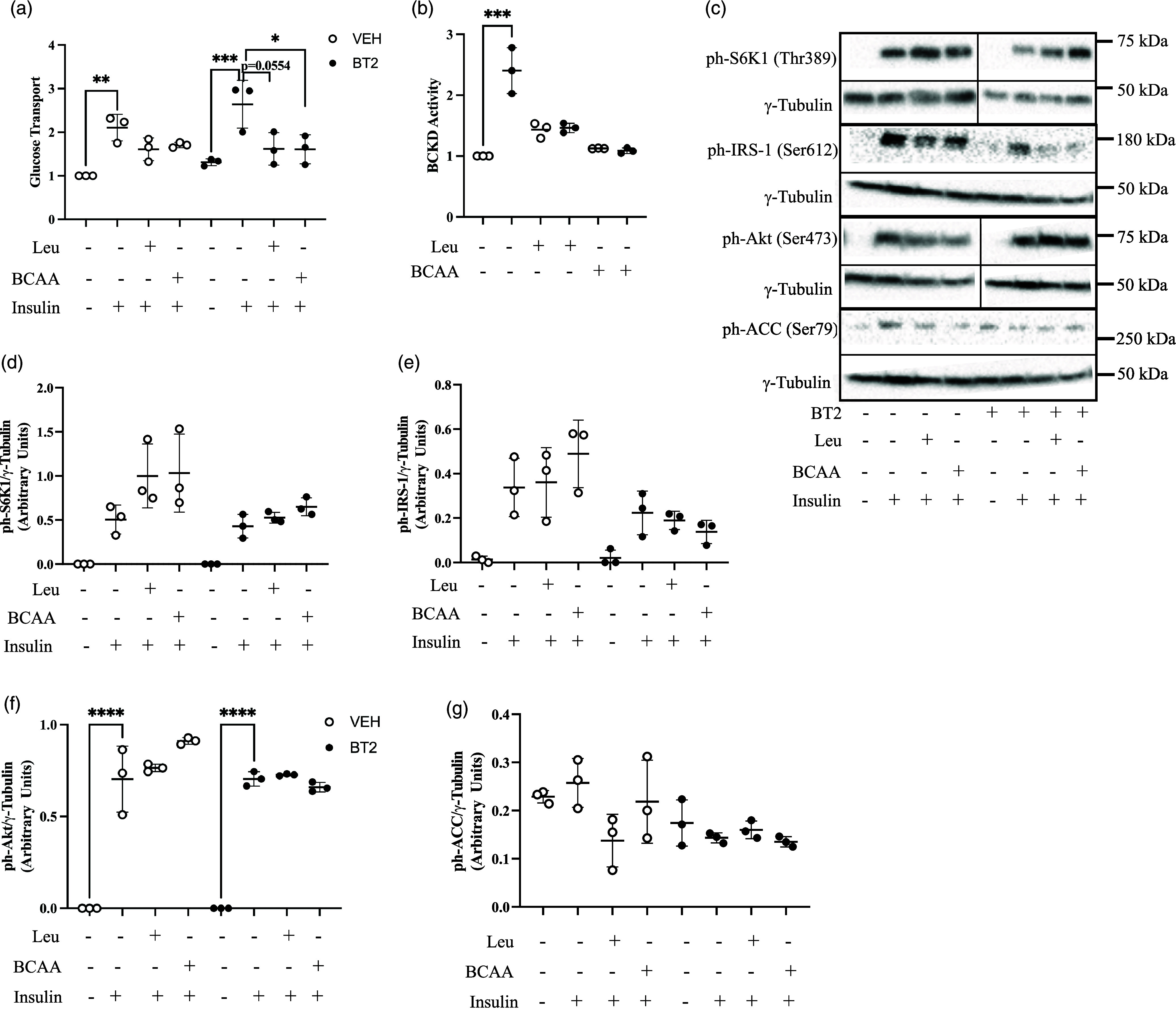
Inhibition of BDK did not modify the effect of BCAA on insulin-stimulated glucose transport. L6 myotubes were incubated for 3 h in 250 μM of BT2 in a starvation medium that lacked amino acids and serum. Incubation then continued in starvation medium with BT2 along with the addition of leucine (150 μM) or BCAA (total 400 μM: consisting of 175 μM of valine, 150 μM of leucine and 75 μM of isoleucine) for 30 min. After, cells were incubated with or without 100 nM insulin for 20 min. Glucose transport (a) and BCKD activity assays (b) were then performed. Proteins in lysates were immunoblotted against ph-S6K1^Thr389^ (c, d), ph-IRS-1^Ser612^ (c, e), ph-Akt^Ser473^ (c, f), and ph-ACC^Ser79^ (c, g). Proteins for western blot were normalised to γ-tubulin as the loading control. Glucose transport was normalised to the no insulin (–insulin) group in the VEH condition. n = 3 biological experiments with 3 technical replicates per experiment. Data are presented as Means ± SD. * P < 0.05, ** P < 0.01, *** P < 0.001, **** P < 0.0001.

**Fig. 4.**
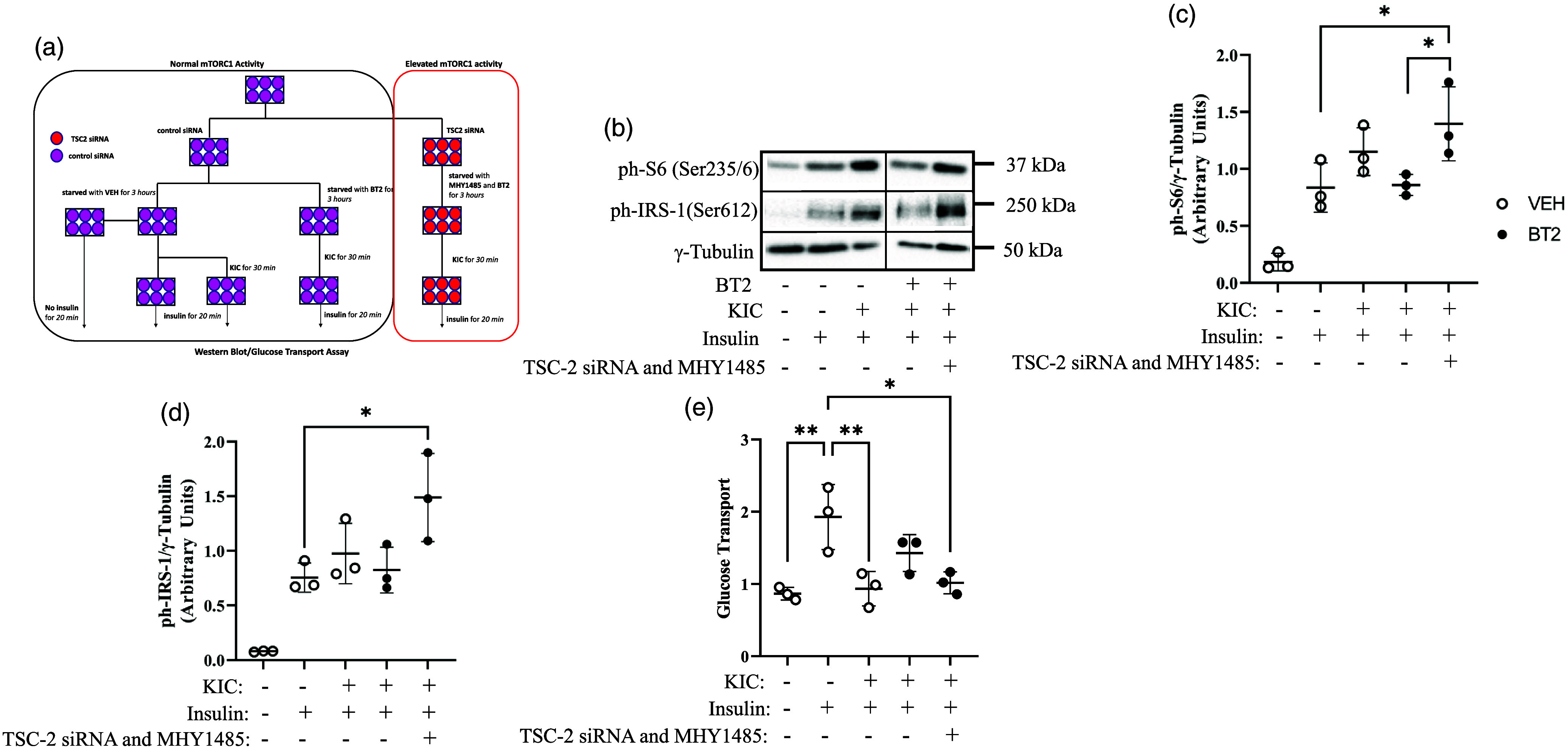
Suppression of mTORC1 is required for the effect of BT2 on glucose transport. mTORC1 was activated with the use of TSC-2 depletion and mTOR activator MHY1485. (a) Cells were transfected with control (purple) or TSC-2 (red) siRNA oligonucleotides. Forty-eight h later, they were treated with or without 250 μM of BT2 and with or without 10 μM of MHY1485 in serum- and amino acid-free medium for 3 h. Afterwards, the cells were supplemented without (−KIC) or with 200 μM KIC (+KIC) for 30 min followed by incubation with or without 100 nM insulin for 20 min. After treatments, proteins in lysates were immunoblotted against ph-S6^Ser235/6^ (b, c) and ph-IRS-1^Ser612^ (b, d). Proteins for western blot were normalized to γ-tubulin as the loading control. Glucose transport assay was performed (e). Glucose transport was normalised to the no insulin (–insulin) group in the VEH condition. Data are presented as Means ± SD; n = 3 biological replicates with 3 technical replicates per experiment. * P < 0.05, ** P < 0.01.

**Fig. 5.**
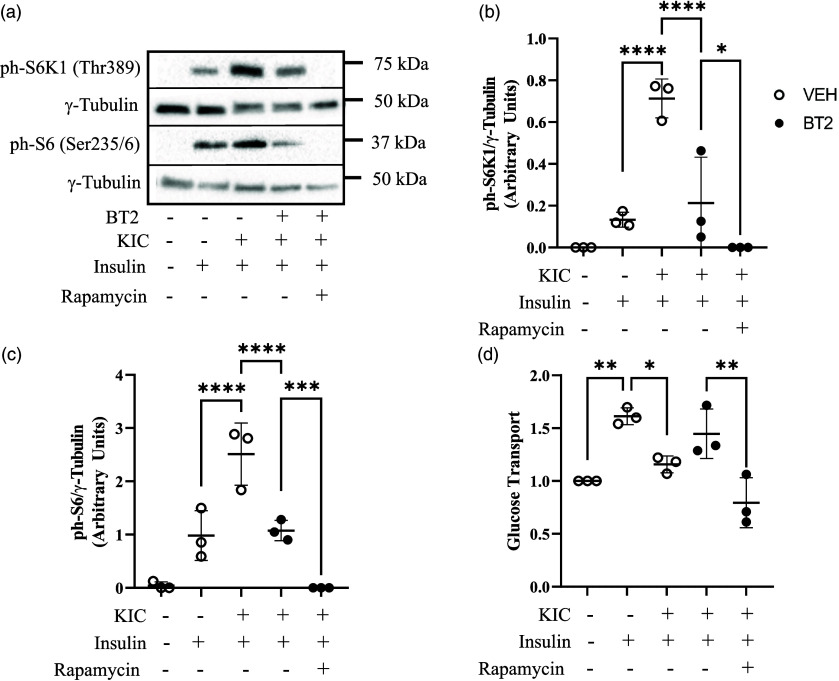
Treatment with rapamycin impairs the effect of KIC-BT2 treatments on insulin-stimulated glucose transport. L6 myotubes were incubated for 3 h in 250 μM of BT2 in a starvation medium that lacked amino acids and serum. Incubation then continued in the medium with BT2 or vehicle that was also supplemented with or without KIC (200 μM) for 30 min and with or without 50 nM rapamycin. After, cells were incubated with or without 100 nM insulin for 20 min. Proteins in cell lysates were then immunoblotted against ph-S6K1^Thr389^ (a, b) and ph-S6^Ser235/6^ (a, c). Proteins for western blot were normalized to γ-tubulin as the loading control. Cells underwent a glucose transport assay (d). Glucose transport was normalised to the no insulin (–insulin) group in the VEH condition. n = 3 biological replicates with 3 technical replicates per experiment. Data are presented as Means ± SD. * P < 0.05, ** P < 0.01, ***, P < 0.001, **** P < 0.0001.

Myotubes were treated with BCAA or BCKA for 30 min. They were then treated with or without 100 nM insulin and the treatment of BCAA/BCKA continued with insulin for 20 minutes. For KIC (Figs. 1–2 and 4–5), the concentration used was 200 μM. For leucine (Fig. 3), the concentration was 150 μM. For the BCKA treatment (Fig. 2), 200 μM was used, made up of 76 μM of KIC, 62 μM of KMV and 62 μM of KIV. For the BCAA treatment (Fig. 3), 400 μM was used, made up of 175 μM of valine, 150 μM of leucine, and 75 μM isoleucine. We used 200 μM of KIC as this concentration induces insulin resistance in L6 myotubes,^(6,7)^ which also prompted us to use a similar value for total BCKAs supplementation. We used 150 μM of leucine as this concentration reduces glucose transport in L6 myotubes the most compared to higher concentrations.^(7)^ The values used for the individual BCAAs are similar to values that are observed in plasma.^(21,26)^

**Fig. 6.**
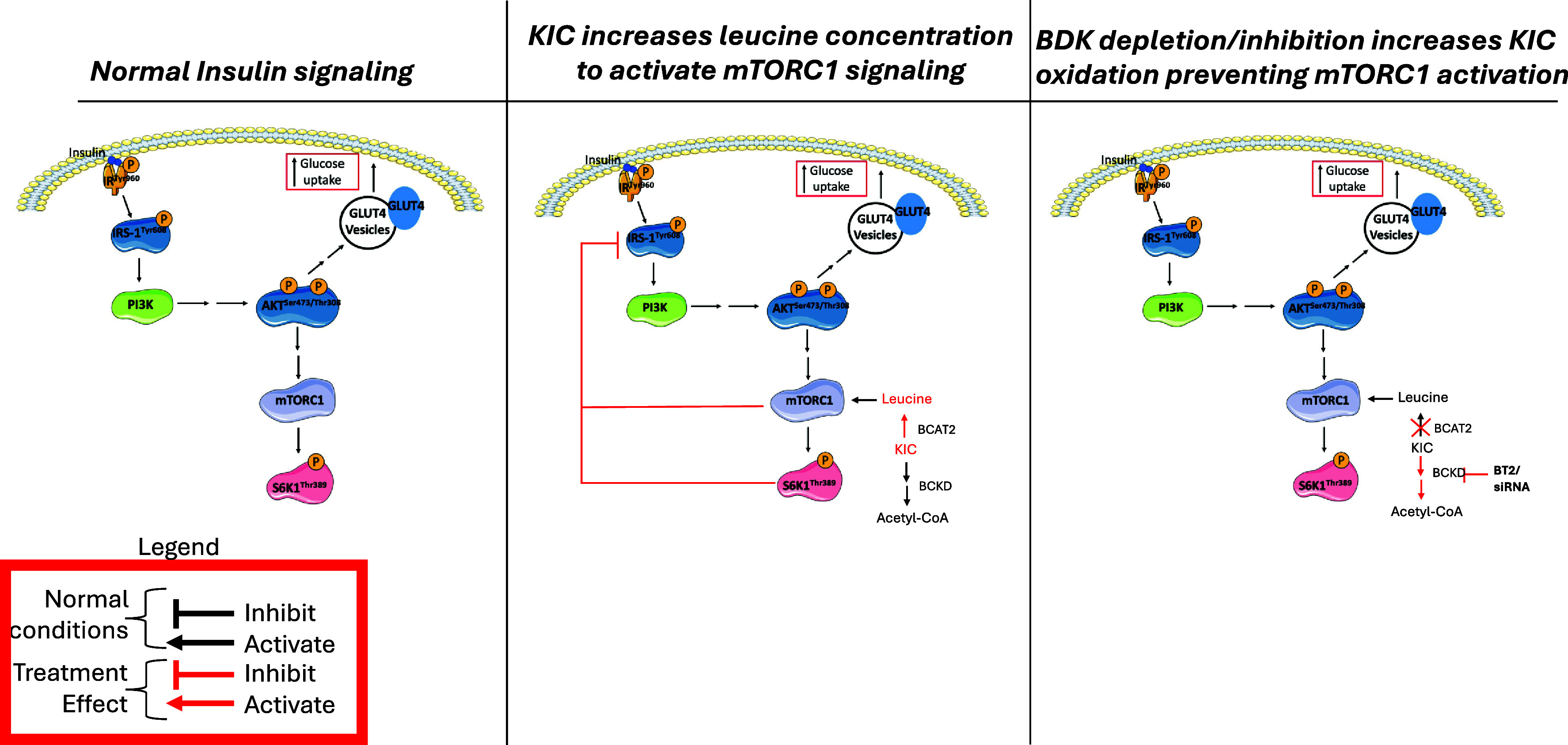
Schematic of the relationship between BCAA/BCKA catabolism and regulation of glucose transport in myotubes. In the first panel, a simplified insulin signalling pathway is shown. In the second panel, with KIC supplementation, KIC is converted back to leucine by BCAT2, which then activates mTORC1/S6K1, leading to the phosphorylation of IRS-1^Ser612^ and thus hindering IRS-1 signalling downstream. In the third panel, with BDK depletion or BT2 treatment, BCKD activity is increased leading to reduced leucine levels, and corresponding attenuation of mTORC1 activation and rescuing of insulin-stimulated glucose transport.

### Gene silencing

On D3 of differentiation, myotubes were transfected with 50 nM scrambled (control, SCR) or BDK (sense 5’-CUAUGCAUGGCUUUGGCUU, anti-sense 5’-GAUACGUACCGAAACCGAA) or TSC-2 (sense 5’-GAGAUUGUUCUGUCCAUAA, anti-sense 5’-CUCUAACAAGACAGGUAUU). Where indicated, cells were treated with 50 nM scrambled or BCAT2 (sense 5′-GAGUGCAUCCGCCAGCUCA, anti-sense 5′-UGAGCUGGCGGAUGCACUC) siRNA oligonucleotides. Transfection was done using Lipofectamine RNAiMAX reagent according to the manufacturer’s instructions.

### BT2 treatment

3,6-dichlorobenzo[b]thiophene-2-carboxylic acid (BT2) is an inhibitor of BDK, a protein kinase that is a negative regulator of BCKD.^(27,28)^ A study that used a range between 160 and 750 μM of BT2 showed that 250 μM reduced ph-BCKD (Ser 293) the most in muscle cells.^(5)^ On D5 of differentiation, L6 myotubes were treated with 250 μM of BT2 in a starvation medium (free of amino acids and serum) for 3 h. Incubation then continued in starvation medium with BT2 or vehicle that was also supplemented with or without KIC (200 μM) for 30 min. After, incubation continued with or without BT2 and KIC. Cells were then incubated with or without 100 nM insulin for 20 min followed by glucose transport assay, BCKD activity assay, or harvesting for immunoblotting and high-performance liquid chromatography (HPLC) analyses. These treatments were done similarly in BDK-depleted cells (Fig. S4).

### mTORC1 activation/inhibition experiments

To examine the contribution of mTORC1 to KIC-induced suppression of insulin-stimulated glucose transport, we initially used TSC-2-depleted cells to activate mTORC1. However, since insulin already diminishes TSC-2 inhibition of mTORC1, the increase in mTORC1 activation from TSC-2 knock-down was only marginally greater in insulin-treated cells (data not shown). Therefore, to effectively activate mTORC1 signalling under the conditions being tested, we combined TSC-2 depletion with treatment of the cells with 4,6-dimorpholino-*N*-(4-nitrophenyl)-1,3,5-triazin-2-amine (MHY1485), an mTOR activator.^(29)^ Myotubes depleted of TSC-2 were treated with vehicle or 10 μM of MHY1485 in a starvation medium for 3 h (Fig. 4a). Incubation then continued in starvation medium with MHY1485 that was also supplemented with or without KIC (200 μM) for 30 min. Myotubes were then incubated with or without 100 nM insulin for 20 min followed by glucose transport assay, or cells were harvested and processed for immunoblotting.

To suppress mTORC1 activity, we used rapamycin as an mTORC1 inhibitor,^(30)^ as done previously in our work.^(7)^ First, cells were starved with or without BT2 (250 μM) for 3 h. They were then treated with or without BT2 in the presence of KIC (200 μM), and with or without rapamycin (50 nM) for 30 min. Cells were then incubated with or without 100 nM insulin for 20 min with or without BT2, KIC, and rapamycin. They were then used for glucose transport assay or harvested for immunoblotting.

### Glucose transport

Following treatments, myotubes cultured in 12-well plates were washed twice with HEPES [4-(2-hydroxy-ethyl)piperazine-1-ethanesulfonic acid]-buffered saline]. They were then incubated in 300 μl of transport solution (HEPES buffer, pH 8, 10 μM 2-deoxyglucose, 0.5 μCi/ml [^3^H]-2-deoxyglucose) for 5 min at 37°C and 2-deoxyglucose transport was performed as described previously.^(7,31)^ Following the 5-min incubation, plates were placed on ice and cells were washed with ice-cold saline 3 times. They were then lysed with 1 ml of cold 0.05 M NaOH. An aliquot of the lysate was used to determine protein concentration while another aliquot was counted. Glucose transport was calculated and expressed as pmol of deoxyglucose transported per μg protein as described before.^(6)^ These values were then normalised to the no-insulin group.

### Amino acid concentrations

Amino acid concentrations were determined as previously described.^(6)^ Briefly, following treatments, myotubes were washed 2× in PBS, and then harvested with 10% trichloroacetic acid. Lysates were centrifuged at 10000 ***g*** for 15 min. Supernatant containing free amino acids were neutralised in a 1:2:1:8 ratio (sample: potassium phosphate buffer: 0.1 N hydrochloric acid: HPLC-grade water, respectively). Neutralised samples were pre-column derivatized with a 1:1 ratio of sample to o-Phthalaldehyde (29.28 mM). They were then injected into a YMC-Triart C18 column (C18, 1.9 μm, 75 × 3.0 mm) fitted onto an ultra-high-pressure liquid chromatography (UHPLC) system that was connected to a fluorescence detector (excitation: 340 nm; emission: 455 nm). Amino acids were eluted with a gradient solution derived from 20 mM potassium phosphate buffer (6.5 pH) (mobile phase A) and a solution made from 45% acetonitrile, 40% methanol and 15% HPLC-grade water (mobile phase B) at a flow rate of 0.8 ml/min. We used a gradient of 5–100% of mobile phase B over 21 min. Amino acid concentrations were calculated using amino acid standard curves and were normalised to total protein that was measured using the Pierce bicinchoninic acid (BCA) protein assay kit.

### BCKA concentrations

Protocol was adapted from a previous study.^(32)^ Following treatments, myotubes were washed 2× in PBS, then harvested with a lysis buffer (1 mM ethylenediaminetetraacetic acid (EDTA), 2% sodium dodecyl sulfate (SDS), 25 mM Tris-HCl pH 7.5, 10 μl/ml protease inhibitor cocktail, 10 μl/ml phosphatase inhibitor cocktail, 1 mM dithiothreitol (DTT)). Lysates were centrifuged at 10000 ***g*** for 10 min. The resulting supernatant was diluted in a 1:2:1:8 ratio (sample: potassium phosphate buffer: 0.1 N hydrochloric acid: HPLC-grade water, respectively). Diluted samples were treated with a 1:1 ratio of a 1,2-diamino-4,5-methylenedioxybenzene (DMB) solution (13.32 mM), sodium sulfite (38.88 mM), 2-mercaptethanol (1 M), HCl (0.696 M) in ddH_2_O). Once samples were treated with DMB, this solution was heated at 85^o^C for 45 minutes and then cooled on ice for at least 5 min. Samples and identically processed standards were injected into an Inertsil ODS-4 column (2 μM, 100 × 2.1 mm; GL Sciences, Torrance, CA, USA) fitted onto an ultra-high-pressure liquid chromatography system (Nexera X2, Shimadzu, Kyoto, Japan) that was connected to a fluorescence detector (Shimadzu, Kyoto, Japan; excitation: 367 nm; emission: 446 nm). Mobile phases were: (A) HPLC-grade methanol/ddH_2_O (30/70, v/v), and (B) HPLC-grade methanol. Gradient elution was performed as follows: 0 min 0% B, 3.33 min 0%B, 5 min 50%B, 17.34 min 50%B. The flow rate was 0.2 ml/min, and the column temperature was maintained at 40^o^C. BCKA concentrations were normalised to total protein as described above for the amino acids.

### BCKD activity assay

BCKD catalyses the irreversible oxidative decarboxylation of the BCKA. Following treatments, each well was treated with 200 μl of starvation media and 856 μL of Krebs Ringer Buffer (0.018 M NaHPO_4_, 0.68% (w/v) NaCl, 0.045% (w/v) KCl, and 0.03% (w/v) MgSO_4_) that was supplemented with 1 mg of thiamine hydrochloride. Then, 61 μl of the reaction mixture (unlabelled valine (18.5 mM) and 1-^14^C labelled valine (3.7 μM) in PBS) was added to this. Wicks soaked with 60 μl of 2 M NaOH were taped hovering above each well. CO_2_ released from the oxidative decarboxylation reaction was captured in 2 M NaOH-soaked filter paper wicks. This radiolabelled bicarbonate on the filter paper wick was transferred into a 20 ml scintillation vial containing 3.5 ml of scintillation fluid and counted. BCKD activity was calculated by dividing the radioactivity counts by the specific activity of valine in the reaction mixture (pmol). This value was then divided by the protein concentration in each well to obtain pmol/μg protein. These values were normalised to the vehicle control group.

### Western blot

Following treatments, cells were processed as described previously.^(33,34)^ Briefly, cells were harvested in lysis buffer (1 mM EDTA, 2% SDS, 25 mM Tris-HCl, pH 7.5, 1 mM DTT, and 10 μl/ml of each of protease inhibitor and phosphatase inhibitor cocktails). Proteins were separated on 10% SDS-polyacrylamide gel electrophoresis (SDS-PAGE) followed by transfer onto polyvinylidene difluoride (PVDF) membranes (0.2 μm pore size). Transfer efficiency was checked with a Ponceau S incubation. This dye was then washed off with three 5-minute washes of Tris Buffered Saline with Tween (TBST). Next, membranes were incubated for one hour in 5% non-fat milk in TBST at room temperature to block non-specific antigen binding. Subsequently, they were quickly washed 3 times, 5 minutes each with TBST at room temperature and then incubated overnight at 4°C with the primary antibody of interest. Primary antibodies used for western blot include ph-S6K1, ph-IRS-1, ph-Akt, ph-S6, ph-ACC, ph-BCKD, BDK, and BCAT2. Proteins for western blot were normalised to γ-tubulin as the reference protein. Following the overnight incubation in primary antibody, membranes were washed 3 times for 5 minutes each with TBST and were incubated in a secondary antibody for three hours at room temperature. Secondary antibodies were diluted into a 5% milk with TBST solution before incubation with the membranes. HRP-conjugated secondary antibodies (anti-rabbit or anti-mouse) were used at 1:10000 dilution in 5% non-fat milk in TBST. Subsequently, membranes were washed 3 times for 5 minutes each with TBST before HRP chemiluminescence substrate was applied. Bio-Rad ChemiDoc XRS+ was used for signal visualisation and the images were quantified with Image Lab software (version 8).

### Data presentation and statistical analysis

Glucose transport data was normalised to the no-insulin group. BCKD activity data are normalised to the vehicle control group. Data are presented as means ± SD. One-way analysis of variance was used and Tukey’s post-hoc tests were done to measure statistically significant differences among means. Significance was determined as P < 0.05. Statistical analyses were performed using GraphPad Prism software (GraphPad, Boston, MA).

## Results

### BDK depletion rescues suppression of insulin-stimulated glucose transport and S6K1/IRS-1 phosphorylation by KIC

BDK depletion (Fig. 1a) increased insulin-stimulated glucose transport (Fig. 1b, P < 0.05). KIC suppressed insulin-stimulated glucose transport, but this was attenuated in BDK-depleted cells (Fig. 1b).

KIC increased insulin-stimulated S6K1 (Thr389) phosphorylation (Fig. 1c and d, P < 0.05), but this increase was attenuated in BDK-depleted cells (Fig. 1c and d). Similarly, KIC increased insulin-stimulated IRS-1 (Ser612) phosphorylation (P < 0.05), a negative regulator of insulin signalling, but this increase was attenuated in BDK-depleted cells (Fig. 1c and e). There was no effect of BDK depletion or KIC supplementation on insulin-stimulated Akt (Ser473) phosphorylation (Fig. 1c and f). These data suggest that the effect of BDK depletion in attenuating the suppressive effect of KIC on insulin-stimulated glucose transport is linked to its effect on S6K1/IRS-1 phosphorylation.

### BDK inhibition rescues BCKA-induced suppression of insulin-stimulated glucose transport

As an alternative, non-genetic approach, we used a relatively more acute pharmacological treatment to increase BCKD activity by incubating the cells with BT2, a BDK inhibitor. BT2 had no effect on myotube morphology and integrity (Supplementary Fig. S1a–d).

As expected, KIC treatment increased intracellular levels of KIC (Fig. S2a, P < 0.001) and leucine (Fig. S2b, P < 0.05), which were attenuated by BT2 treatment. Incubation with KIC reduced insulin-stimulated glucose transport. As was observed in BDK-depleted cells, the effect of KIC on insulin-stimulated glucose transport was largely attenuated with BT2 treatment (Fig. 2a). We also wanted to assess what the effect of all the BCKA would be on insulin resistance since intracellularly, KIC exists in the context of the other BCKA. BCKA treatment only marginally increased KIC (Fig. S2a), leucine (Fig. S2b), KIV (Fig. S2c), and KMV (Fig. S2d) levels. These increases were attenuated by BT2 treatment. As was observed for KIC, incubation with the BCKA reduced insulin-stimulated glucose transport but this effect was attenuated in BT2-treated cells (Fig. 2a). The effect of KIC or BCKA occurred in parallel with reduced phosphorylation of BCKD-E1α especially in the presence of BT2 (Fig. 2b and c) and the tendency for increased BCKD activity with BT2 treatments (Fig. 2d). BCKA and KIC increased insulin-stimulated S6K1 (Thr389) phosphorylation (Fig. 2b and e), which was attenuated with BT2 treatment. There was no effect of KIC and BCKA, with or without BT2, on IRS-1 (Ser612), although the values in the BT2 groups were smaller across the different ketoacid treatment groups (Fig. 2b and f). Whether or not co-treated with BT2, KIC/BCKA did not modify phosphorylation of Akt (Ser473, Fig. 2b and g) or acetyl-CoA carboxylase (ACC, Ser79) (Fig. 2b and h), a protein downstream of BDK.^(19)^ Thus, the effect of BT2 in attenuating the suppressive effect of KIC/BCKA on insulin-stimulated glucose transport is likely mediated by the action of BDK on BCKD but not on other proteins downstream of BDK. As shown in our previous study,^(7)^ the conversion of KIC to leucine requires BCAT2 (Fig. S3a and b and Fig. 6, right panel).

BT2 and KIC co-treatment in BDK-depleted cells did not further enhance glucose transport compared to KIC treatment in BDK-depleted cells (Fig. S4a and b). This suggests that BT2 and BDK depletion acts along a common pathway (i.e. BCKD) and that increased glucose transport in response to BT2 is due to the inhibition of BDK and not to potential off-target effects of BT2 (Fig. S4a and b).

### BCAA supplementation has no effect on insulin-stimulated glucose transport with or without BT2

We wondered how the effect of KIC/BCKA on glucose transport would compare with that of leucine/BCAA since the pairs are connected. In general, leucine or the BCAA had small suppressive effect on glucose transport, with only the effect of BCAA being significant and only in the presence of BT2 (Fig. 3a). Leucine/BCAA had no effect on BCKD activity (Fig. 3b) but tended to increase S6K1 phosphorylation (40% increase relative to insulin only; Fig. 3c and d). BT2 treatment also tended to suppress leucine and/BCAA mediated increases on S6K1 (∼32% decrease relative to control; Fig. 3c and d) and IRS-1 serine phosphorylation (∼70% decrease relative to control; Fig. 3c and e). Insulin-stimulated increase in Akt (Ser473) phosphorylation was not modified by leucine, BCAA or BT2 treatment (Fig. 3c and f). There were no treatment effects on the phosphorylation of ACC (Ser79) (Fig. 3c and g), although the effect of insulin on ACC phosphorylation tended to be lower (∼50%) in BT2-treated cells.

### mTORC1 activation abolishes the effect of BT2 on KIC-induced suppression of insulin-stimulated glucose transport

Our data in Fig. 1c–e linked the suppression of insulin-stimulated glucose transport to the activation of S6K1/IRS1 axis. Data from our studies with BDK knock-down/inhibition also suggest that the restoration of glucose transport in the presence of KIC is linked to the S6K1/IRS1 signalling axis. To experimentally establish that this signalling axis is critical to the suppressive effect of KIC, we combined TSC-2 deletion and treatment with MHY1485 (an mTORC1 activator) to restore the mTORC1/S6K1/IRS-1 axis in the context of BDK inactivation and KIC treatment (Fig. 4a–d). As shown before, KIC suppressed insulin-stimulated glucose transport, which was attenuated by BT2. However, this attenuation is lost when mTORC1/S6K1/IRS-1 is restored (Fig. 4e) showing that the inhibitory mTORC1 phosphorylation of IRS-1 contributes at least in part to the suppressive effects of KIC on insulin-stimulated glucose transport in myotubes.

### The action of BT2 in KIC-treated cells requires mTORC1 activity

In our previous work, the suppressive effect of KIC was dependent on mTORC1, as rapamycin treatment attenuated the suppressive effect of KIC on insulin-stimulated glucose transport.^(7)^ We were curious to examine if this suppression held true with BT2 co-treatment. As expected, rapamycin co-treatment with BT2 and KIC further reduced S6K1 (Fig. 5a and b, P < 0.05) and S6 phosphorylation (Fig. 5a and c, P < 0.001). Surprisingly, this led to further suppression of insulin-stimulated glucose transport (Fig. 5d, P < 0.01). These data suggest that although increased mTORC1 activation reduces glucose transport, some mTORC1 activity might be needed to facilitate glucose transport, underlying the complexity of mTORC1 signalling and its link to glucose transport.

## Discussion

In this study with myotubes, we demonstrated a specific suppressive effect of KIC and other branched-chain keto acids in modulating insulin-stimulated glucose transport and phosphorylation (and by implication, the activity) of related signalling proteins. Using both genetic and pharmacological interventions, we showed that activation of steps downstream of KIC (BCKA) in the BCAA catabolic pathway attenuated the suppressive effect of KIC (Fig. 6). Significantly, we established a role for mTORC1/S6K1/IRS-1 signalling axis in the insulin resistance caused by KIC (Fig. 6). Together, these data demonstrate a role for KIC and the other BCKA in the development of insulin resistance in skeletal muscle and that mechanisms that increase flux through BCKD could be beneficial in preventing/correcting insulin resistance in skeletal muscle. Given the significance of skeletal muscle in whole-body insulin activities (glucose, amino acid, and fatty acid disposal)^(35)^ and that muscle insulin resistance is a primary underlying cause for T2DM,^(25)^ data presented here also imply that interventions that increase BCAA catabolic flux hold promise for prevention/management of T2DM and its sequelae.

In previous studies, mTORC1 mediates the suppressive effects of BCAA^(36,37)^ and BCKA^(6,38)^ on insulin resistance. Activated mTORC1/S6K1 phosphorylates inhibitory serine residues of IRS-1 (Ser612)^(39)^ hindering signalling downstream.^(1,15)^ Many of the preceding studies have either focused on the effect of the BCAA at the whole body level,^(40–42)^ or did not study BCKA, so the significance of KIC and the other BCKA in mediating muscle insulin resistance was not clear. Here, we showed that KIC activated the S6K1-IRS-1 feedback loop, but BDK depletion or inhibition attenuated this, most likely by increasing the catabolism of KIC. Consistent with this, leucine level was reduced in cells in which BDK action was inhibited (Fig. S2b).

Elevated levels of plasma BCAA and BCKA are linked to future diabetes,^(43)^ which is mediated by insulin resistance.^(44)^ Lotta *et al.* showed that a genetic predisposition to impaired BCAA metabolism is implicated in T2DM.^(45)^ Also, BCAA oxidation and levels of BCAA catabolic enzymes in skeletal muscle are reduced in insulin-resistant states.^(16,18)^ We have also demonstrated that depletion of the E1α subunit of BCKD reduces insulin-stimulated glucose transport.^(6)^ Consistent with these findings, activating BCKD via BT2 treatment improves whole-body insulin sensitivity, liver steatosis and fatty acid oxidation in the liver of obese mice. However, liver-specific knock-down for BDK in these obese mice did not yield similar results, suggesting other tissues like skeletal muscle may play a role in BT2-mediated benefits on insulin sensitivity.^(23)^ Some of the previous studies on BCKD activity have focused on non-skeletal tissues such as liver and adipose tissue because of the believe that BCKD activity in the muscle is low. If this were so, KIC or BCKA treatment should have minimal effect on muscle insulin sensitivity. Our previous study^(6)^ and data presented here clearly implicate muscle BCKD activity as an important node of metabolic derangement that can contribute to insulin resistance. Activating BCKD by sodium phenylbutyrate treatment for two weeks improved insulin sensitivity in T2DM patients.^(24)^ However, Blair *et al.* demonstrate that although increasing BCKD activity in skeletal muscle reduces plasma BCAA, this is not sufficient to improve insulin sensitivity. Additionally, increasing BCKD activity in the liver and skeletal muscle does not improve insulin sensitivity but treatment with BT2 at the whole-body level does, suggesting contribution of tissues other than skeletal muscle and liver.^(21)^ In another twist, Zhao *et al.* show that metformin improved insulin sensitivity, but worsened BCAA catabolism^(46)^ and that AMP-activated protein kinase (AMPK) phosphorylation was increased, along with a decrease in BCKD activity and increased BCAA/BCKA levels.^(46)^ The increase in AMPK activity, an mTORC1 inhibitor,^(47)^ could prevent BCAA input to mTORC1 activation. This scenario of improved insulin sensitivity in the context of elevated BCAA suggests the existent of alternative inputs that may influence the physiological outcome of BCAA-mTORC1-glucose transport interactions.

Exercise is an example of such an input. Daily exercise is a determinant of insulin sensitivity,^(48)^ as exercise training increases glucose uptake, and GLUT4 protein expression in skeletal muscle,^(49–51)^ even in T2DM patients.^(52,53)^ BCAA levels are increased in T2DM patients^(2,3)^ and yet an acute bout of exercise reduces blood glucose levels in T2DM patients similar to non-diabetic individuals.^(54)^ Interestingly, in diabetic mice, 4 weeks of exercise reduced plasma BCAA levels while increasing insulin sensitivity.^(55)^ Additionally, endurance exercise increases BCAA catabolism and BCKD activation in skeletal muscle of humans^(56)^ and rats.^(57,58)^ Also, endurance training reduces the amount of BDK bound to the BCKD complex in rat skeletal muscle and liver.^(58–61)^ Thus, the effects of exercise are similar to those of BT2, as the increase in BCAA oxidation from exercise may offset the effects of BCAA and their dysregulated metabolism in insulin-resistant states.

Our data showing that activating mTORC1 in the presence BT2 re-established the suppression of glucose transport by KIC is consistent with our previous publication in which rapamycin (an mTORC1 inhibitor) treatment attenuated the suppressive effect of KIC on insulin-stimulated glucose transport.^(7)^ However, our data in the current study, which examined rapamycin treatment in BT2 and KIC-treated cells suggests that the action of BT2 requires some mTORC1 activity because abolishing mTORC1 activity reduced insulin-stimulated glucose transport. Interestingly, rapamycin reduces insulin-stimulated glucose transport by reducing insulin-stimulated GLUT4 translocation.^(62)^ Shorter treatment (2 weeks) of rapamycin reduced insulin sensitivity while 6-week treatment showed no change, and a longer (20 week) treatment improved insulin sensitivity.^(63)^ Additionally, muscle-specific ablation of raptor (an essential mTORC1 component) in mice worsens glucose tolerance,^(64)^ while in another study muscle-specific knockout of raptor worsens insulin tolerance.^(65)^ Furthermore, rapamycin inhibits FK506 binding protein 12 (FKBP12), a component of mTORC1. FKBP12 is required for ryanodine receptor function, which are intracellular calcium channels involved in excitation-contraction coupling.^(66)^ Loss of FKBP12 proteins leads to calcium leak, which suppresses insulin sensitivity in skeletal muscle *in vitro* and *in vivo.* ^(67)^ Thus, reductions in glucose transport from rapamycin could be due to off-target effects. To prevent off-target effects of the drug, it would be better to knock-down raptor as a way of inhibiting mTORC1, but as stated above, muscle-specific raptor ablation in mice worsened insulin sensitivity.^(64,65)^ Ultimately, this suggests that mTORC1 activation must be kept at a certain threshold and any divergence from this may impact insulin sensitivity.

BCAA supplementation in mice with muscle-specific BDK knockout enhances S6K1 phosphorylation,^(68)^ which suggests that BCAT2 (required for converting BCAA to BCKA which are the substrates for BCKD) might be limiting for BCAA catabolism even with induced BCKD activity. This is in line with previous data that show that BCAT2 is required for insulin resistance induced by KIC,^(7,69)^ as BCAT2 knock-down reduces the conversion of KIC back to leucine.^(6)^ Overall, our data with BDK inhibition, alone or in the context of concurrent mTORC1 activation, clearly demonstrates that the activation of mTORC1/S6K1/IRS-1 axis is at least in part causative for the suppressive effect of KIC on insulin-stimulated glucose transport in myotubes. However, our data as well as other studies cited above suggest that there is a requirement for some mTORC1 activity for induction of muscle glucose transport.

Akt is a key intermediate in insulin-stimulated glucose transport.^(70)^ However, while we observed an insulin effect on Akt Ser473 phosphorylation in control myotubes, neither KIC nor BT2 had an effect on Akt Ser473 phosphorylation. Zhou *et al.* demonstrated that BCAA treatment in ob/ob mice fed a low protein diet reduced Akt (Thr308) phosphorylation in skeletal muscle, and BT2 increased Akt (Thr308) phosphorylation in ob/ob mice.^(22)^ They also demonstrated that BCKA (500 μM) treatment in 3T3-L1 cells reduced Akt (Thr308) phosphorylation, and this was dependent on mTORC1.^(22)^ Conversely, in one study with women, whey protein ingestion or consumption of an equivalent amount of leucine had no effect on Akt phosphorylation (Ser473 or Thr308) even though ingestion of whey protein suppressed glucose transport.^(71)^ Thus, while many studies implicate Akt activation in insulin signalling to glucose transport, this is not universal. For example, others have reported elevated level of Akt phosphorylation in apparent cases of leg muscle insulin resistance^(72–74)^ or in response to inflammation,^(75)^ implying that the relationship between Akt phosphorylation and insulin-stimulated glucose transport may be context-specific, and that impairment in insulin-stimulated glucose transport by KIC may be mediated at steps distal to Akt.

It is interesting to note apparent differences in the effect of BCAA relative to BCKA on glucose transport. The suppressive effects of BCKA on insulin-stimulated glucose transport appear more pronounced than what was observed with BCAA. Also, the attenuating effects of BT2 are more apparent in BCKA- vs BCAA-treated cells. The apparent higher potency of KIC/BCKA in inhibiting insulin-stimulated glucose transport might arise from the fact that KIC/BCKA by themselves on one hand, and the leucine/BCAA that can be derived from transamination of KIC/BCKA on the other hand, may each activate mTORC1/S6K1, leading to higher activation of S6K1/IRS1 axis and as a consequence a greater suppression of glucose transport. This hypothesis is supported by the higher activation of S6K1 in BCKA-treated cells (compare Figs. 2e to 3d).

A limitation of this work is that it is an *in vitro* study. We have also focused on BCAA/BCKA to the exclusion of other amino acids, their metabolites, and other nutrients. The presence of those other nutrient variables would likely affect the data obtained. Nevertheless, our study examining the effect of increasing BCKD activity on insulin sensitivity in myotubes allows identification of mechanisms of effects of BCKA in a manner that might not be possible in an *in vivo* setting. Also, we used 200 μM of KIC, a value that is higher than 20–30 μM for each of the BCKA in obese mice.^(46)^ However, other studies used 500 μM-25 mM KIC.^(5,22,76)^ Future studies will need to examine lower concentrations of the BCKA.

In conclusion, we showed that inhibition/depletion of BDK in L6 myotubes attenuated the suppression of insulin-stimulated glucose transport that was induced by BCKA via mechanisms involving the mTORC1/S6K1/IRS-1 signalling axis. Given the significance of skeletal muscle in whole-body insulin-induced nutrient utilisation and that impaired insulin action in skeletal underlies or worsens a number of diseases,^(77)^ our data suggest that interventions that can improve BCAA oxidation flux, especially downstream of BCKA, hold promise for prevention/treatments of insulin resistance and its associated human chronic diseases.

## Supporting information

Mann and Adegoke supplementary material Mann and Adegoke supplementary material

## Abbreviations

**ab-am:** antibiotic-antimycotic; **ACC:** acetyl-CoA carboxylase; **Akt:** protein kinase B; **AMEM:** alpha modification of eagle’s medium; **BCAA:** branched-chain amino acids; **BCAT2:** branched-chain aminotransferase 2; **BCKA:** branched-chain ketoacids; **BCKD:** branched-chain ketoacid dehydrogenase; **BDK:** BCKD kinase; **BT2:** 3,6-dichlorobenzo[b]thiophene-2-carboxylic acid; **DM:** differentiation medium; **DMB:** 1,2-diamino-4,5-methylenedioxybenzene; **DMSO:** dimethyl sulfoxide; **DTT:** dithiothreitol (DTT); **D5:** day 5; **FBS:** foetal bovine serum; **FKBP12:** FK506 binding protein 12; **GM:** growth medium; **HEPES:** 4-(2-Hydroxyethyl)piperazine-1-ethanesulfonic acid; N-(2-Hydroxyethyl)piperazine-N′-(2-ethanesulfonic acid); **HS:** horse serum; **IRS-1:** insulin receptor substrate-1; **KIC:** ketoisocaproic acid; **KMV:** α-keto-β-methylvaleric acid; **KIV:** α-ketoisovaleric acid; **mTORC1:** mammalian/mechanistic target of rapamycin complex 1; **MHY1485:** 4,6-dimorpholino-*N*-(4-nitrophenyl)-1,3,5-triazin-2-amine; **OPA:** O-phthalaldehyde; **PBS:** phosphate-buffered saline; **ph:** phosphorylated; **PVDFL:** polyvinylidene difluoride; **Ser:** serine; **siRNA:** small interfering ribonucleic acid; **S6:** ribosomal protein S6; **S6K1:** p70 ribosomal protein S6 kinase-1; **Thr:** threonine; **TSC-2:** tuberculosis sclerosis complex-2; **T2DM:** type 2 diabetes mellitus; **UPHLC:** ultra-high-pressure liquid chromatography.

## Supplementary material

The supplementary material for this article can be found at https://doi.org/10.1017/jns.2024.66
