# Supplementary material for: Elevated BCAA catabolism reverses the effect of branched-chain ketoacids on glucose transport in mTORC1-dependent manner in L6 myotubes
Source: J Nutr Sci. 2024 Oct 18;13:e66. doi: 10.1017/jns.2024.66 (PMC11503859; doi:10.1017/jns.2024.66)
Supplement: Mann and Adegoke supplementary material [file S2048679024000661sup001.pdf]

Elevated BCAA catabolism attenuates branched-chain ketoacid-induced suppression of glucose transport in mTORC1-dependent manner in L6 myotubes

Gagandeep Mann and Olasunkanmi A. J. Adegoke*

School of Kinesiology and Health Science and Muscle Health Research Centre, York University, Toronto, ON, Canada M3J 1P3

***Corresponding Author: Olasunkanmi A. J. Adegoke** Muscle Health Research Centre, School of Kinesiology and Health Science, York University, 4700 Keele Street, Toronto, ON, Canada.

Tel: 416-736-2100 ext. 20887 Fax: 416-736-5774. Email: oadegoke@yorku.ca

Fig S1

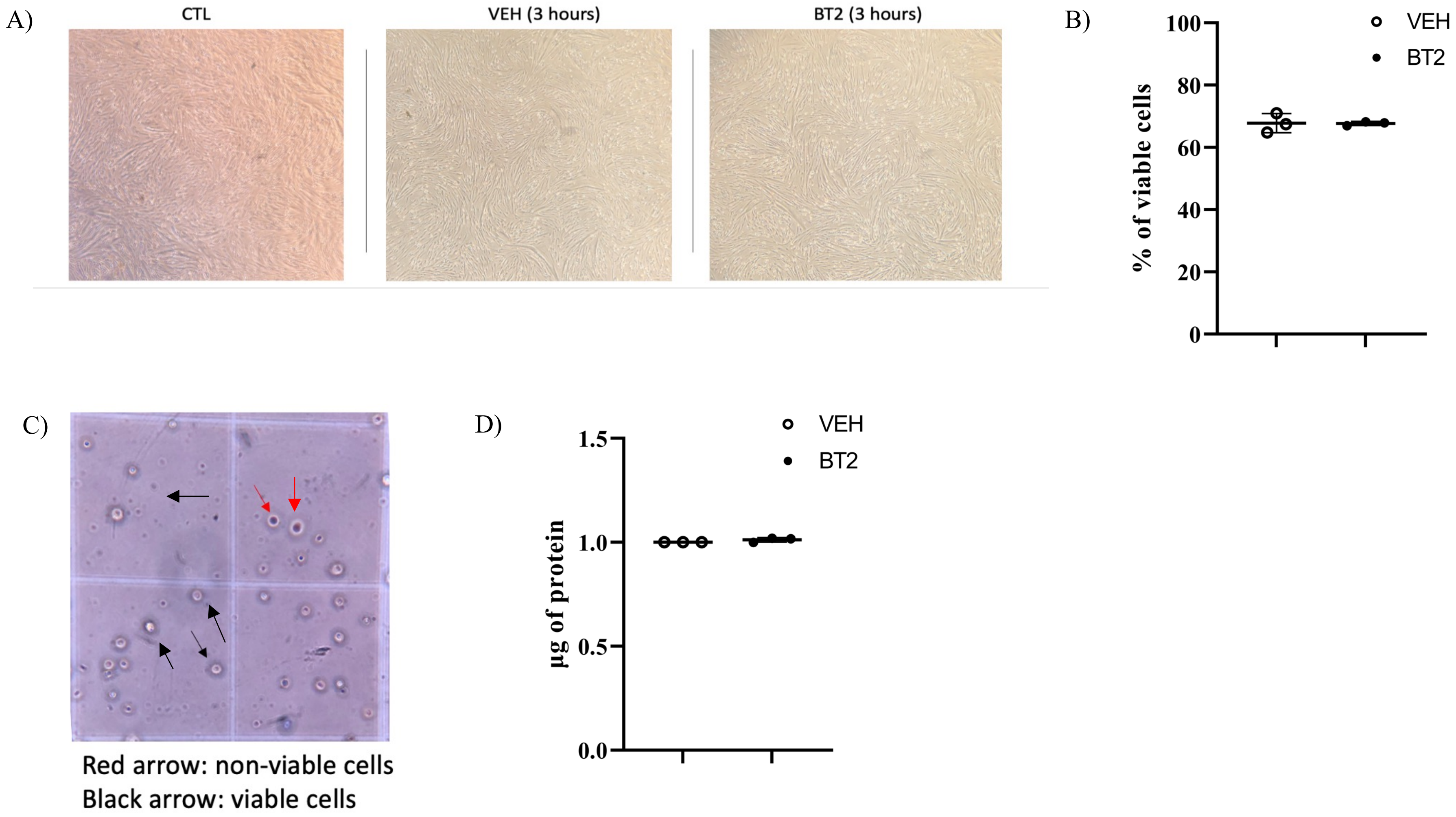

A)
CTL
VEH (3 hours)
BT2 (3 hours)
B)
% of viable cells
100
80
60
40
20
0
VEH
BT2
C)
Red arrow: non-viable cells
Black arrow: viable cells
D)
µg of protein
1.5
1.0
0.5
0.0
VEH
BT2

Fig S2

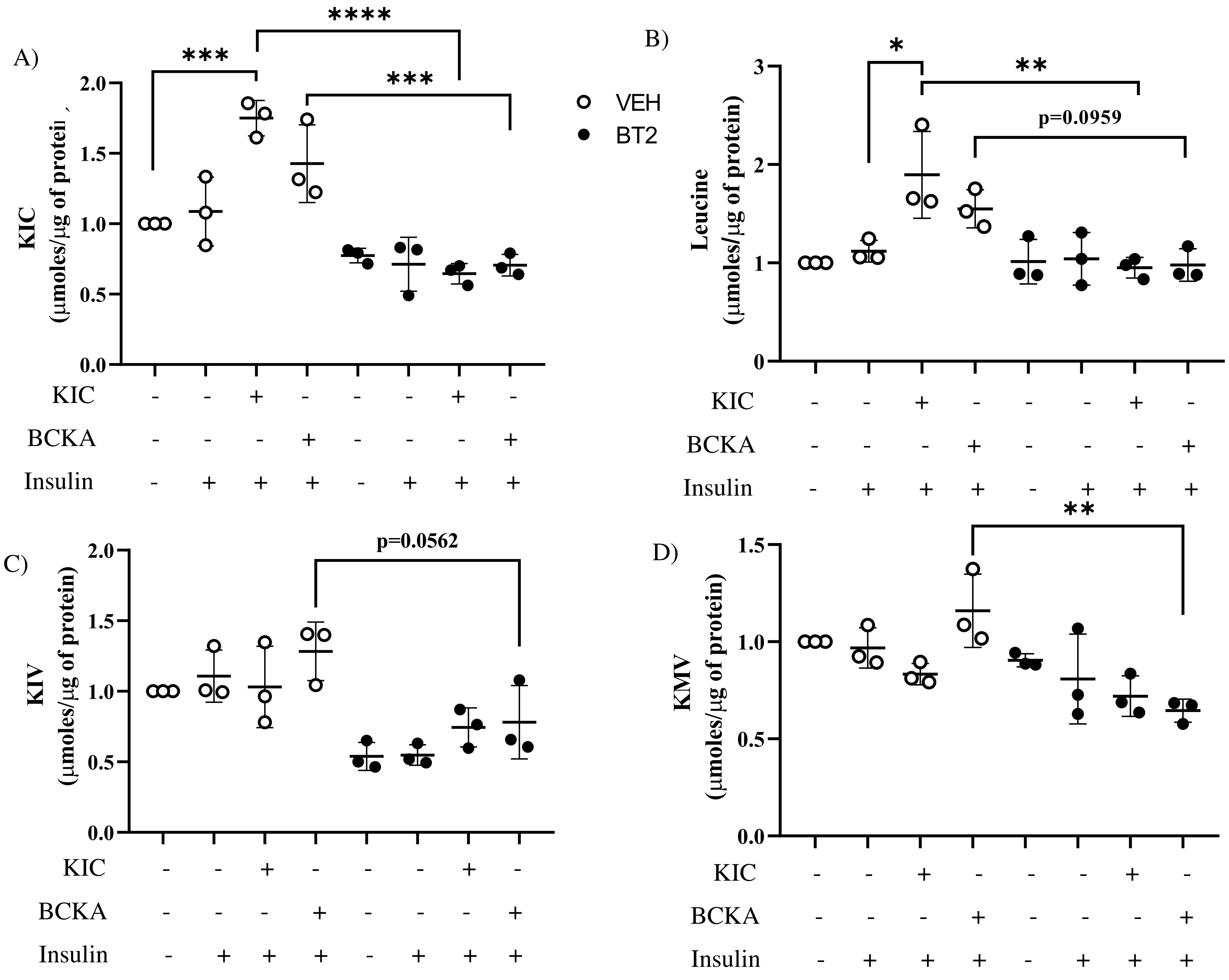
A)
KIC (µmoles/µg of protei
2.0
1.5
1.0
0.5
0.0
***
****
***
VEH
BT2
KIC
BCKA
Insulin
B)
Leucine (µmoles/µg of protein)
3
2
1
0
*
**
p=0.0959
C)
KIV (µmoles/µg of protein)
p=0.0562
D)
KMV (µmoles/µg of protein)
**

Fig S3

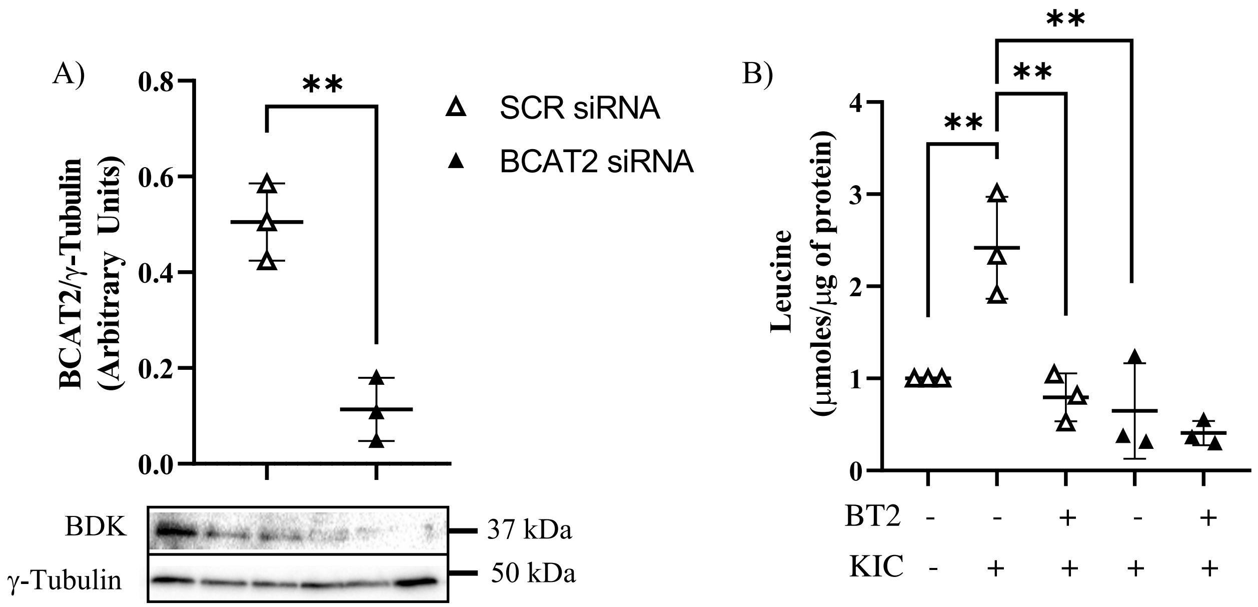

A)
**
SCR siRNA
BCAT2 siRNA
BCAT2/γ-Tubulin (Arbitrary Units)
0.8
0.6
0.4
0.2
0.0
BDK
γ-Tubulin
37 kDa
50 kDa
B)
**
**
**
Leucine (µmoles/µg of protein)
4
3
2
1
0
BT2 - - + - +
KIC - + + + +

Fig S4

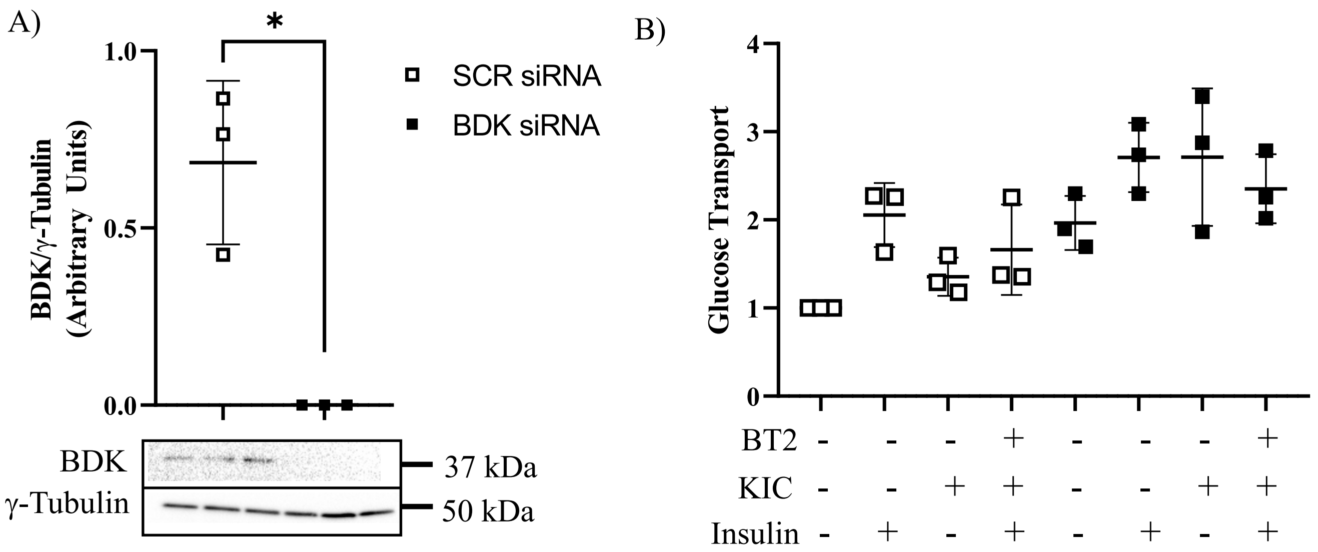
A)
*
1.0
0.5
0.0
BDK/γ-Tubulin
(Arbitrary Units)
SCR siRNA
BDK siRNA
BDK
γ-Tubulin
37 kDa
50 kDa
B)
Glucose Transport
0
1
2
3
4
BT2 - - - + - - - +
KIC - - + + - - + +
Insulin - + - + - + - +
